# Supplementary material for: Initial conditions combine with sensory evidence to induce decision-related dynamics in premotor cortex
Source: Nat Commun. 2023 Oct 16;14:6510. doi: 10.1038/s41467-023-41752-2 (PMC10579235; doi:10.1038/s41467-023-41752-2)
Supplement: Supplementary file 1 — Supplementary Information [file 41467_2023_41752_MOESM1_ESM.pdf]

Supplementary Information

Initial conditions combine with sensory evidence to induce decision-related dynamics in premotor cortex

Pierre O Boucher[1], Tian Wang[1], Laura Carceroni[2], Gary Kane[3], Krishna V Shenoy[4,5,6,7,8,9,10,*], Chandramouli Chandrasekaran[1,3,11,12,†]

[1]*Department of Biomedical Engineering, Boston University, Boston, 02115, MA, USA*
[2]*Undergraduate Program in Neuroscience, Boston University, Boston, 02115, MA, USA*
[3]*Department of Psychological and Brain Sciences, Boston University, Boston, 02115, MA, USA*
[4]*Department of Electrical Engineering, Stanford University, Stanford, 94305, CA, USA*
[5]*Department of Neurobiology, Stanford University, Stanford, 94305, CA, USA*
[6]*Howard Hughes Medical Institute, HHMI, Chevy Chase, 20815-6789, MD, USA*
[7]*Department of Bioengineering, Stanford University, Stanford, 94305, CA, USA*
[8]*Stanford Neurosciences Institute, Stanford University, Stanford, 94305, CA, USA*
[9]*Bio-X Program, Stanford University, Stanford, 94305, CA, USA*
[10]*Department of Neurosurgery, Stanford University, Stanford, 94305, CA, USA*
[11]*Center for Systems Neuroscience, Boston University, Boston, 02115, MA, USA*
[12]*Department of Anatomy & Neurobiology, Boston University, Boston, 02118, MA, USA*

*Deceased
†Corresponding Author

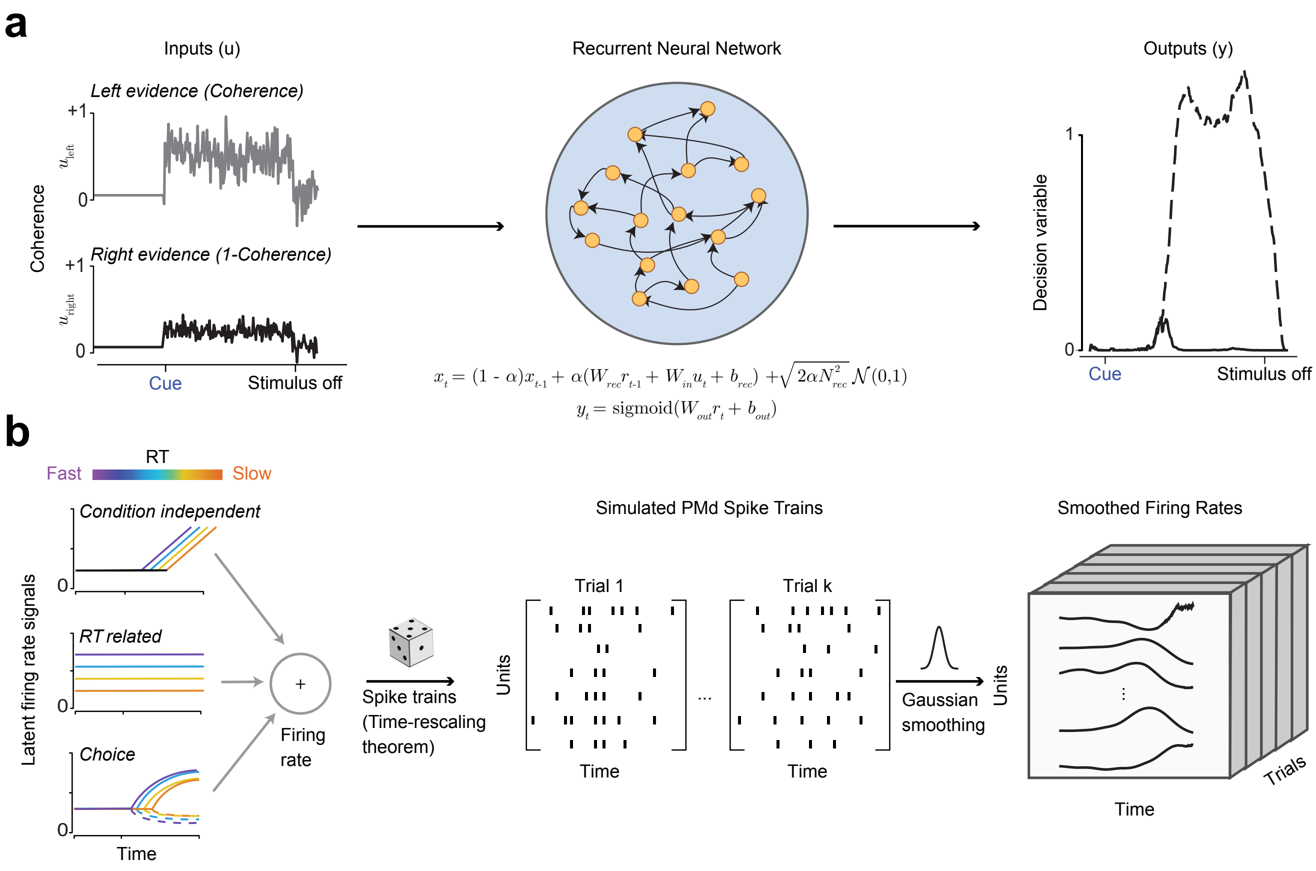

Figure S1: **Schematic illustrating RNN models of decision-making and simulation of hypothetical PMd populations.** (**a**) Schematic of RNN inputs and outputs used to model decision-making. State equations displayed below graphical representation of the RNN. The inputs are the left evidence (given by coherence (Coh)), and right evidence (given as 1-Coh). The network produced two decision-variables for left and right choices respectively and a decision was made when one of these decision-variables reached a threshold of 0.7. (**b**) Our simulations of hypothetical PMd neurons were based on our prior work (Chandrasekaran et al, 2017) that showed that PMd neurons largely increase or decrease their firing rates before checkerboard onset, or respond on or around movement onset. The schematic shows the latent signals that compose an increased neuron's firing rate. The firing rates of these neurons were composed of a condition-independent signal (Kaufman et al, 2016) whose latency varied modestly with RT, a RT-related firing rate signal that was present throughout the trial, and a choice signal whose latency was also modulated with RT. We used these insights and the insight that about 20% of units covary with RT before checkerboard onset (Fig. S17d) to develop a simulation of PMd neural responses during decision-making.

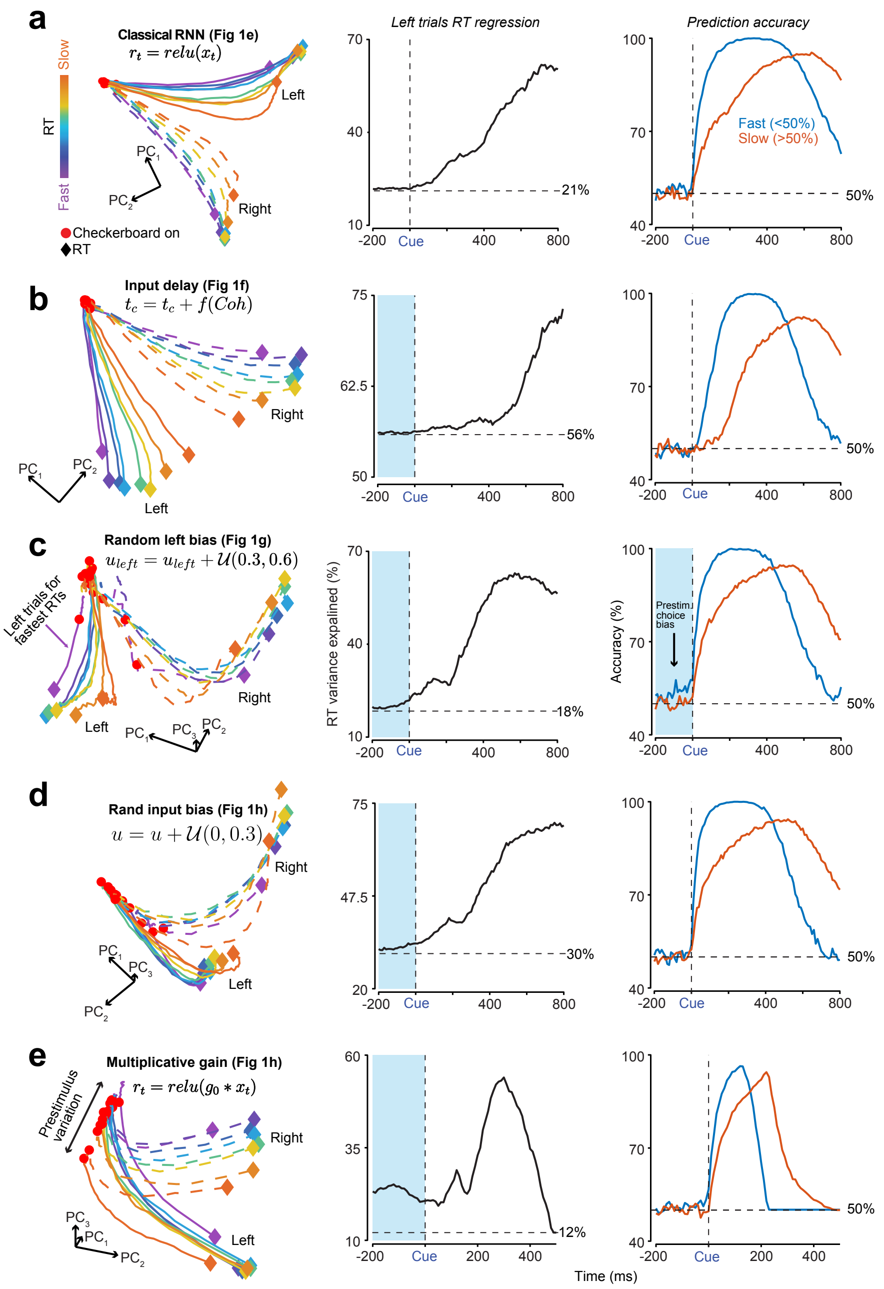

Figure S2: **Recurrent neural network models corresponding to hypotheses in Fig. 1.** (**a-e**) Left: PCA trajectories. Middle: Percent RT variance explained by RNN firing rates. Right: Percent choice-decoding accuracy from RNN firing rates for fast (blue, < 50th percentile for RT) and slow (orange, > 50th percentile for RT) trials of 5 RNN models. Cue on x-axis and vertical black dotted line is checkerboard onset. Horizontal black dotted lines represent the RT variance explained by coherence and chance prediction level, respectively for the middle and right columns. (**a**) Basic RNN model does not show prestimulus dynamics (left), prestimulus neural covariation with RT (middle) or prestimulus choice predictability (right). (**b**) A delay applied to the basic RNN model does not change the prestimulus dynamics (left) but results in delayed neural covariation with RT (middle) and delayed choice selectivity for slow trials (right). (**c**) Applying a random input bias on left trials results in prestimulus neural covariation with RT for left trials (left and middle). A prestimulus choice bias also exists (right). (**d**) Applying a random input bias for both left and right trials results in prestimulus neural covariation with RT (left and middle). A prestimulus choice bias does not exist (right). (**e**) A multiplicative gain applied to recurrent state x changes prestimulus neural state (left) such that there is prestimulus neural covariation with RT (middle) and a minimal prestimulus relationship with choice (right). Source data are provided as a Source Data file.

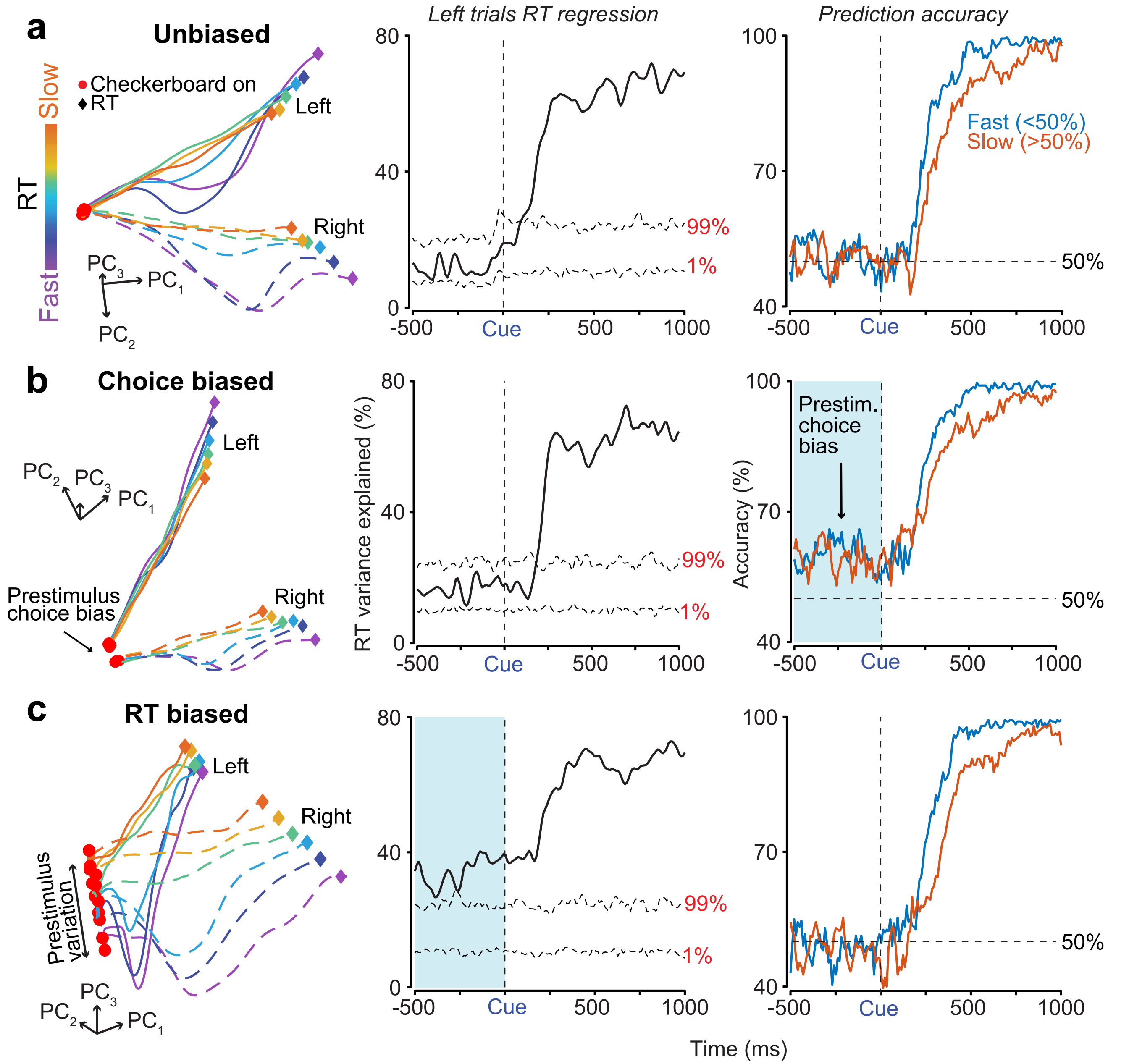

Figure S3: **Dynamics of simulated neural populations with either no bias, choice bias or RT bias.** (**a-c**) Left: PCA trajectories. Middle: Percent RT variance explained by simulated neurons' firing rates. Right: Percent accuracy for choice-decoding results by firing rates of 3 simulation scenarios. Blue lines denote the fastest 50% of trials, and orange the slowest 50% of trials. Cue on x-axis and vertical black dotted line is checkerboard onset. Horizontal black-dotted lines are the 1st and 99th percentile of percent variance explained by 100 shuffles of RT data (middle) or 50% accuracy (right). (**a**) Simulated PMd neurons without prestimulus bias for either RT or choice have minimal prestimulus neural dynamics (left), no prestimulus neural covariation with RT (middle) nor any prestimulus choice predictability (right). (**b**) Simulated PMd neurons with prestimulus choice bias don't show any prestimulus RT variations (middle) but demonstrate prestimulus choice bias (left & right). (**c**) Simulated PMd neurons show clear prestimulus dynamics patterning with respect to RT and covariation (left/middle) but no prestimulus choice selectivity (right). Source data are provided as a Source Data file.

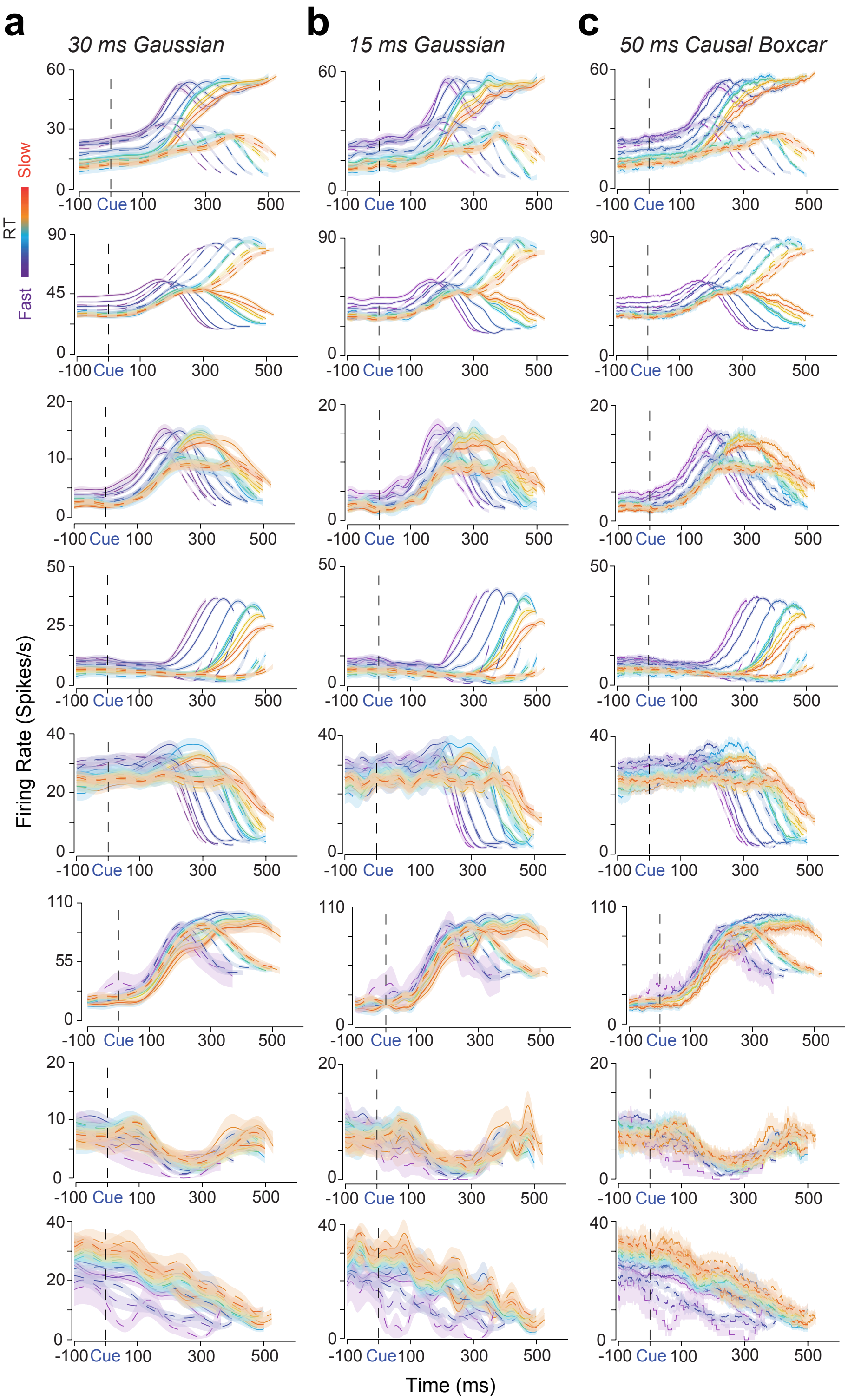

Figure S4: **Firing rates of PMd units largely show the same structure even when smoothed with different kernels.** Firing rates of 8 example units in PMd from monkeys T and O aligned to stimulus onset (right - dashed, left - solid) (Cue/vertical dashed black line) organized by 11 RT bins and both action choices. Firing rates are plotted until the median RT of each color coherence and until the center of each RT bin (e.g., for a 400 to 500 ms RT bin, this would be 450 ms). Color bar indicates RT. Color-matched shading is SEM. First 6 rows of units are the same 6 units from Fig. 3. Reprinted here with original (**a**) 30 ms Gaussian smoothing (**b**) 15 ms Gaussian smoothing and (**c**) 50 ms boxcar smoothing for easy comparison of effects of smoothing filters on spiking activity. Smaller kernels increase noise but the prestimulus covariation with RT is largely consistent and only weakly dependent on smoothing kernel. Source data are provided as a Source Data file.

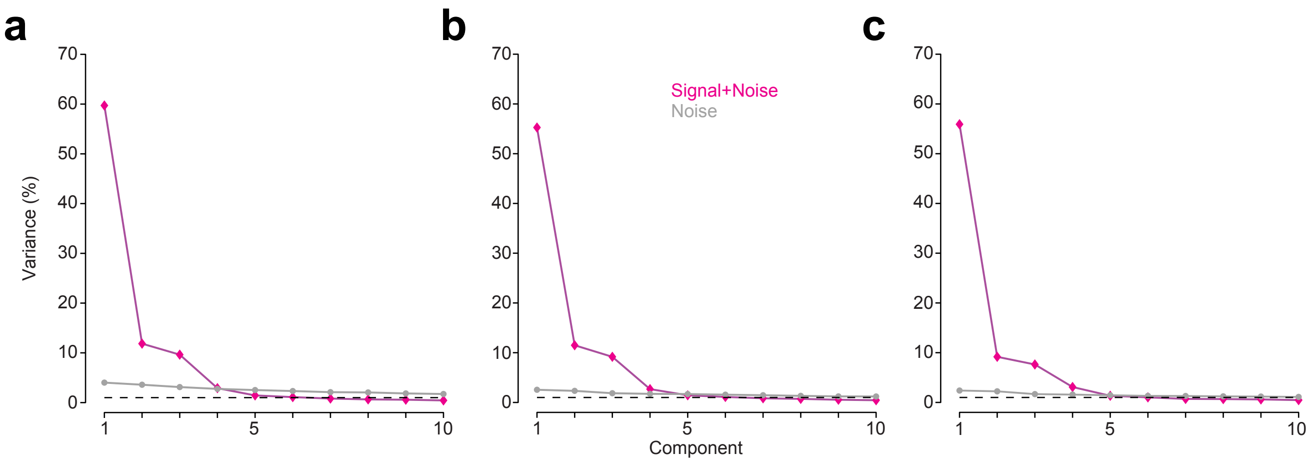

Figure S5: **Percent variance explained by 1st 10 components from PCAs organized by RT and choice.** Signal+noise and noise variance explained by the first 10 components for PCAs organized by RT and choice where different smoothing kernels were used to derive firing rates from spiking activity. (**a**) 30 ms Gaussian smoothing kernel. The first 6 components capture over 90% of the variance. (**b**) 15 ms Gaussian smoothing kernel. The first 6 components capture 87.59% of the variance and the 1st 10 components capture over 90% of the variance. (**c**) 50 ms boxcar smoothing kernel. The first 6 components capture 85.67% of the variance and the first 17 components capture over 90% of the variance. To derive the error bars (not visible) for the signal+noise PCA, we used bootstrapping (50 repeats) over trials. Source data are provided as a Source Data file.

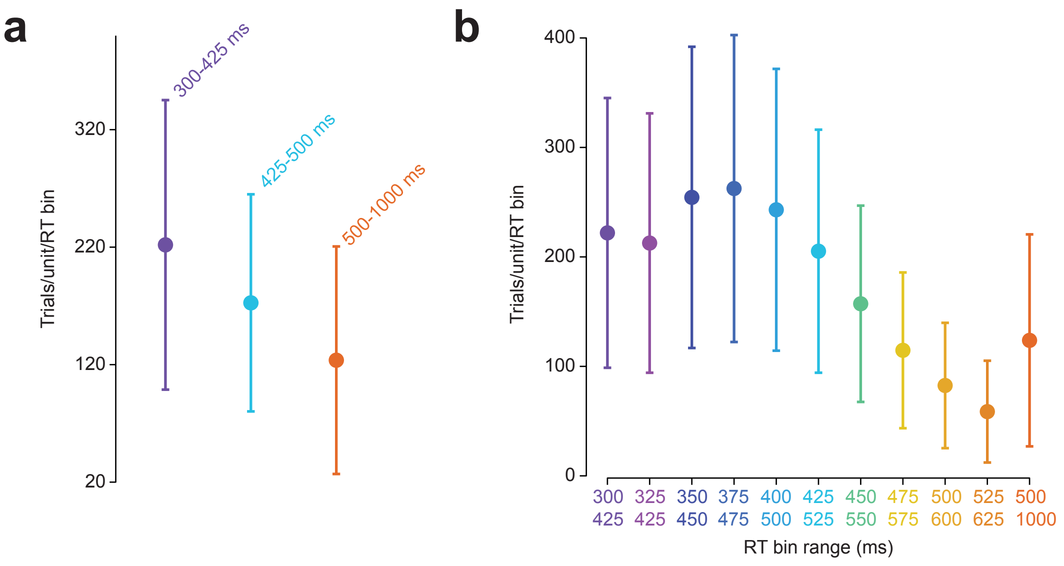

Figure S6: **Average number of trials per unit per RT bin.** Mean number of trials per unit (n = 996) for (**a**) non-overlapping and (**b**) overlapping RT bins (e.g., Fig. 5 and Fig. 4). Error bars are standard deviation around the mean. Source data are provided as a Source Data file.

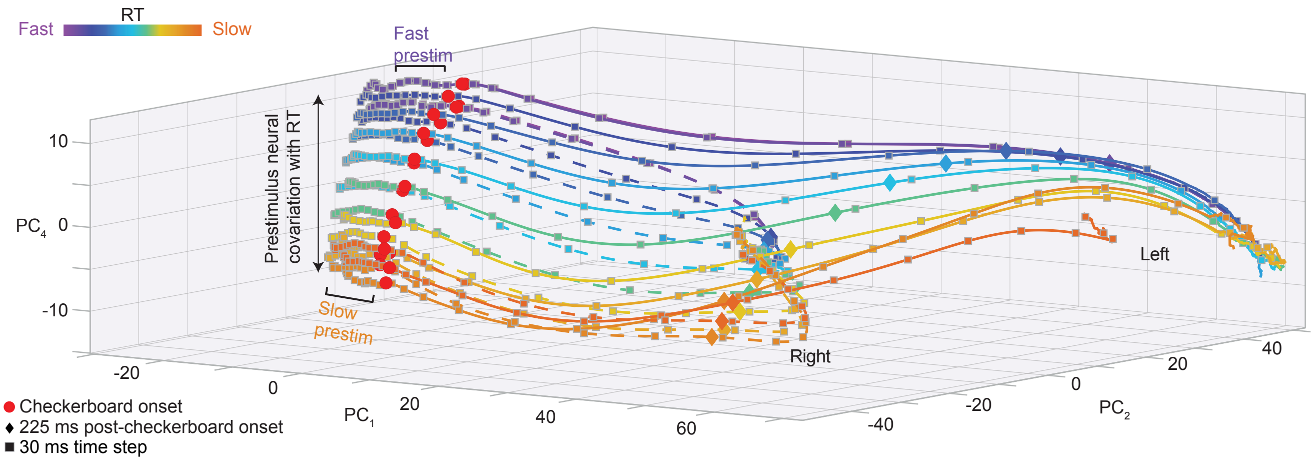

Figure S7: **PC plot with axes equalized for PCs 1, 2, and 4.** State space trajectories of the 1st, 2nd and 4th PCs ($PC_{1,2,4}$) aligned to checkerboard onset (red dots). Prestimulus neural activity robustly separates as a function of RT bin. Diamonds and squares, color matched to their respective trajectories, indicate 225 ms post-checkerboard onset and 30 ms time steps respectively. Faster RT trajectories appear to move faster in the prestimulus period than slower RTs (fast/slow prestim). Axes are equalized to show that PC1 has the largest magnitude followed by PC2 and PC4. This figure is a companion to Fig. 4b. Source data are provided as a Source Data file.

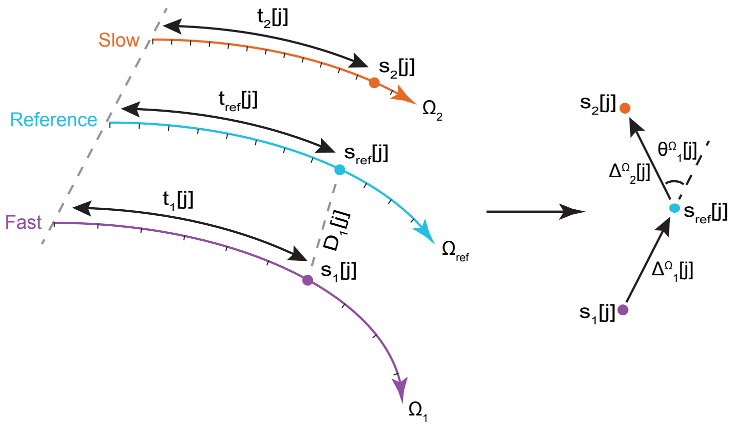

Figure S8: **Diagram of kinematic analysis of neural trajectories (KiNeT)** The middle trajectory in cyan denotes the reference trajectory Ω_{ref}. Two non-reference Ω_1 (violet) and Ω_2 (orange) denote trajectories that evolve faster and slower than Ω_{ref}, respectively. For each timepoint j, the corresponding neural state on the reference trajectory is denoted as $s_{ref}[j]$ and $t_{ref}[j]$ is the corresponding time for the reference trajectory to evolve from the initial point to $s_{ref}[j]$. The closest points to $s_{ref}[j]$ on the fast and slow trajectories as measured by Euclidean distance are denoted by $s_1[j]$ and $s_2[j]$. $t_1[j]$ and $t_2[j]$ are the corresponding times to reach $s_1[j]$ and $s_2[j]$. The vector connecting the two closest points on adjacent trajectories at timepoint j is denoted as $\Delta i^{\Omega}[j]$. The angle between two adjacent vectors is calculated as $\theta_i[j]$. This schematic is drawn based on (Remington et al, 2018).

- i - index of non-reference trajectories
- j - index of timepoints associated with the reference trajectory
- Ω_i - the ith non-reference trajectory
- τ - timepoints associated with the non-reference trajectories
- $\Omega_i(\tau)$ - position of non-reference trajectory at timepoint τ
- $s_{ref}[j]$- position of reference trajectory at the jth timepoint
- $s_i[j]$- closest position of Ω_i to $s_{ref}[j]$ at timepoint j
- $t_i[j]$- the non-reference timepoint for when it's closest to the reference trajectory at timepoint j (corresponding time of $s_i[j]$)
- $D_i[j]$- distance between nearest point on non-reference trajectory Ω_i and reference trajecory at index j
- $\Delta i^{\Omega}[j]$- vector connecting two nearest points on two adjacent trajectories.
- $\theta_i[j]$- angle between two adjacent vectors Δ^{Ω}_i and Δ^{Ω}_{i+1}
- argmin - where function achieves its minimum at point j

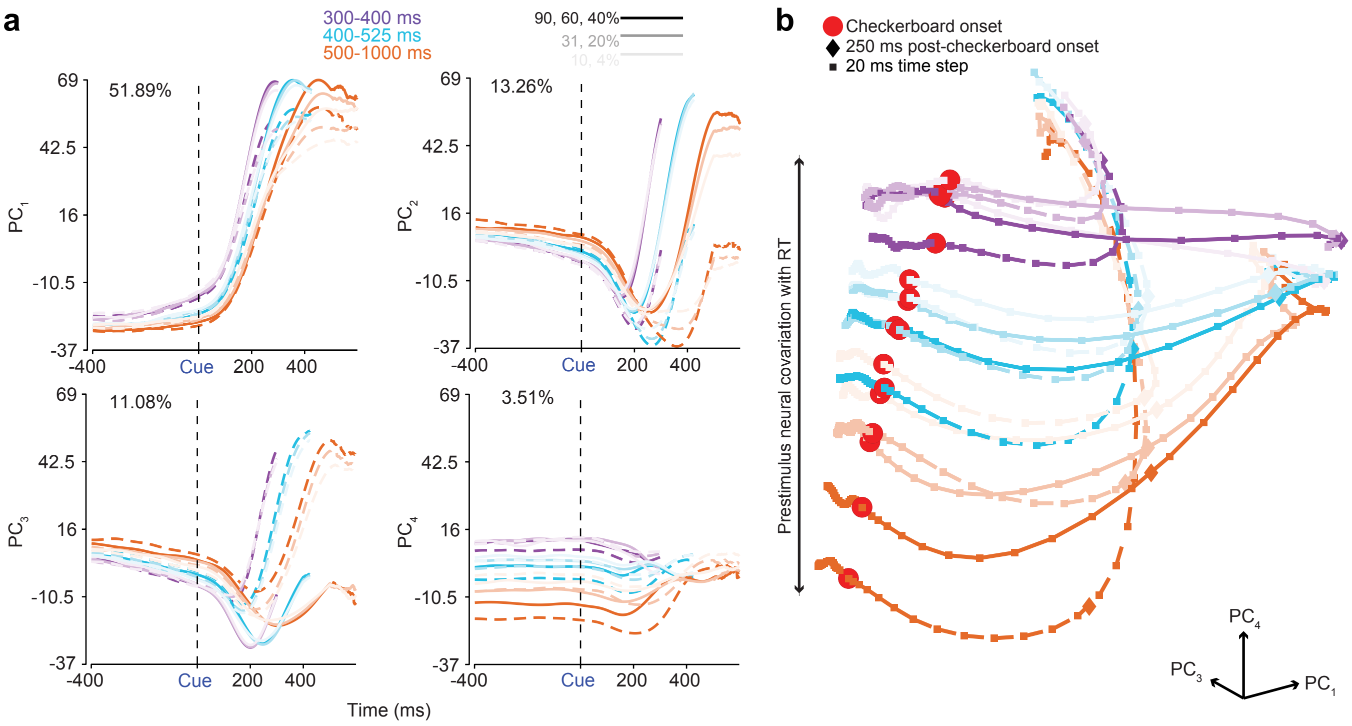

Figure S9: **Combined coherence and RT PCA recapitulates previous findings.** (**A**) The first four PCs ($PC_{1,2,3,4}$) of trial averaged firing rates organized across 3 RT bins (violet - fastest bin to orange - slowest bin and 3 coherence bins from easiest to hardest (darkest opacity to lightest), both reach directions (right - dashed lines, left - solid lines), and aligned to checkerboard onset. Variance explained is indicated at the top of each plot. Note for monkey O in 32 sessions an additional coherence (96%) was used as a stimulus and this is included as one of the 'easiest' coherences. (**B**) State space trajectories of the 1st, 3rd and 4th PCs ($PC_{1,3,4}$) aligned to checkerboard onset (red dots). Prestimulus neural activity robustly separates as a function of RT bin. Diamonds and squares, color matched to their respective trajectories, indicate 250 ms post-checkerboard onset and 30 ms time steps respectively. Source data are provided as a Source Data file.

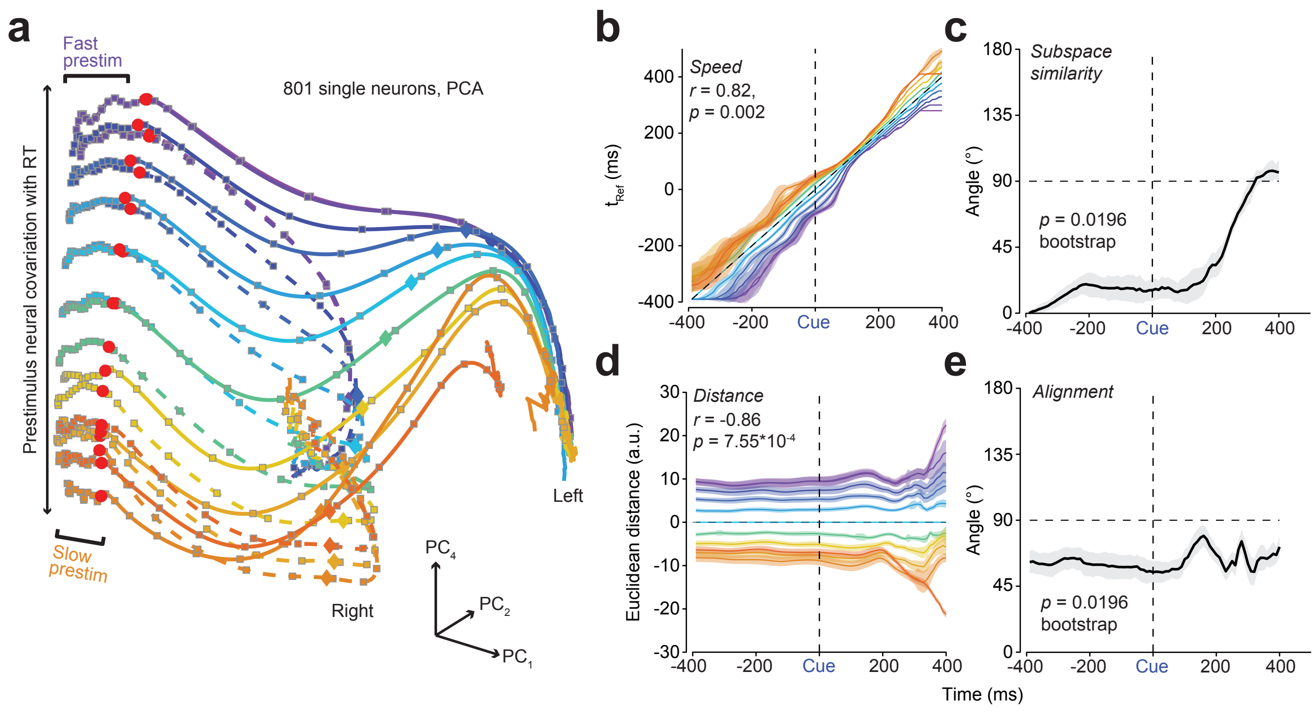

Figure S10: **Replication of PCA and KiNeT findings using only single units (801/996).** (**a**) State space trajectories of the 1st, 2nd and 4th PCs ($PC_{1,2,4}$) aligned to checkerboard onset (red dots). Prestimulus neural activity robustly separates as a function of RT bin. Diamonds and squares, color matched to their respective trajectories, indicate 225 ms post-checkerboard onset and 30 ms time steps respectively. Faster RT trajectories appear to move faster in the prestimulus period than slower RTs (fast/slow prestim). Note axes are deliberately not equalized to better visualize the fluctuations in the initial condition before checkerboard onset. (**b**) KiNeT Time to reference (t_{Ref}, relative time at which a trajectory reaches the closest point in Euclidean space to the reference trajectory) analysis shows that trajectories for faster RTs reach similar points on the reference trajectory (cyan, middle trajectory) earlier than trajectories for slower RTs. This result suggests that the dynamics for faster RTs are closer to a movement initiation state than slower RTs. (**c**) Angle between subspace vector at each timepoint and subspace vector at the first timepoint (-400 ms). The angle between subspace vectors is largely consistent but the space rotates as choice signals emerge (~ 200 ms). (**d**) KiNeT distance analysis showing that trajectories are consistently spatially organized before and after stimulus onset and correlated with RT. (**e**) Average relative angle between adjacent trajectories for each timepoint. The angles between adjacent trajectories were largely less than 90° for the prestimulus period but approach orthogonality as choice signals emerge poststimulus (~ 200 ms). Error bars are color-matched SEM (n = 50 bootstraps) in **b** - **e**. Correlations in **b** & **d** were tested with two-sided t tests. p values presented in **c** & **e** were derived from one-sided 50-repetition bootstrap tests (subspace and average relative angle different from 90°). Source data are provided as a Source Data file.

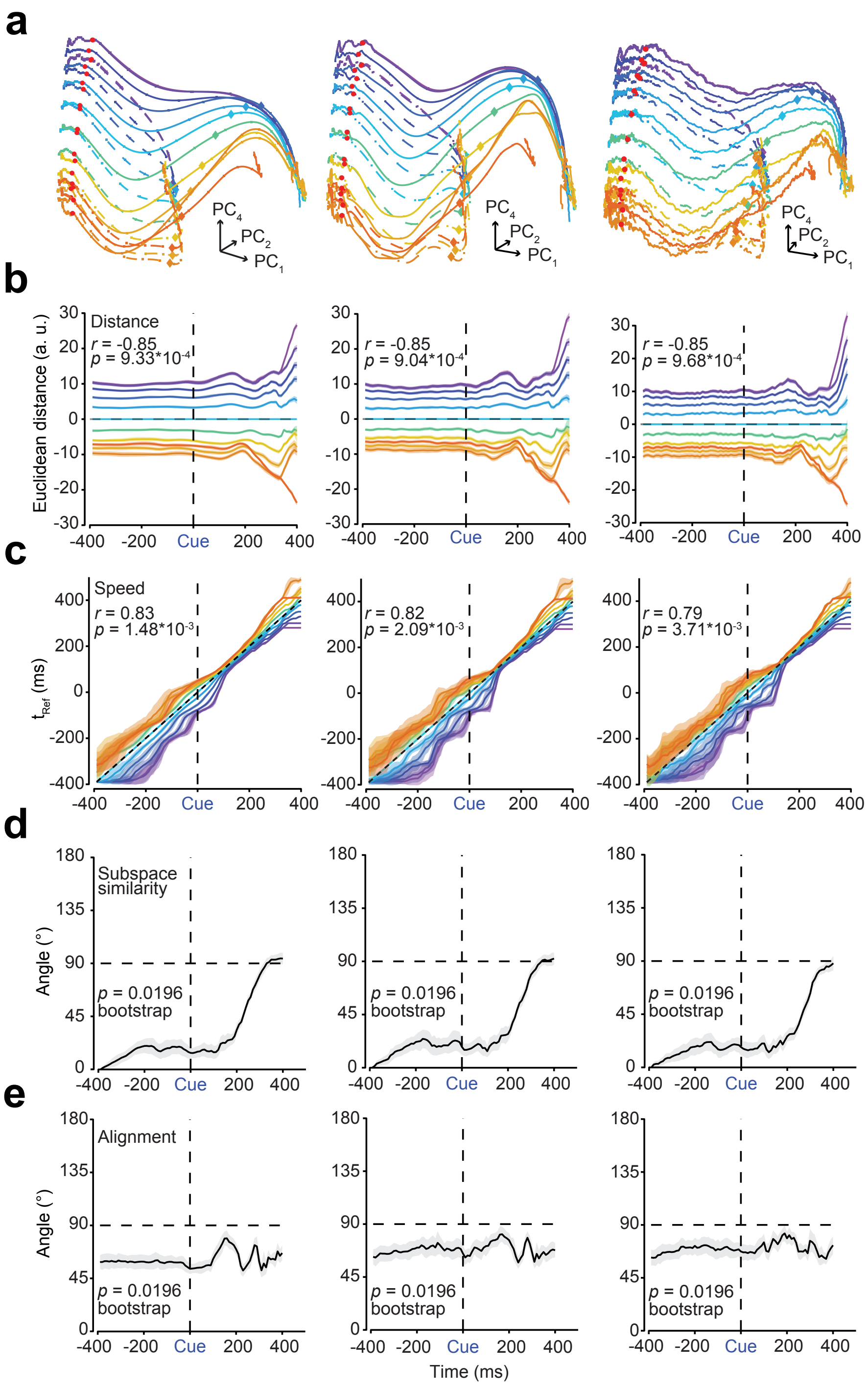

Figure S11: **The major conclusions from this study are not dependent on particular smoothing kernels.** (**a**) 1st, 2nd and 4th PC ($PC_{1,3,4}$) state space plots aligned to checkerboard onset (red dots) where firing rates were smoothed with different smoothing kernels. From left to right 30 ms Gaussian, 15 ms Gaussian, and a 50 ms boxcar. This organization by smoothing kernels is consistent throughout the rest of the figure. (**b**) KiNet distance analysis for the three smoothing kernels. (**c**) KiNet speed analysis organized for the three smoothing kernels. (**d**) KiNet subspace similarity analysis for the three smoothing kernels. (**e**) KiNet alignment analysis for the three smoothing kernels. See Fig. 4 for additional explanations. Error bars are color-matched SEM (n = 50 bootstraps) in **b** - **e**. Correlations in **b** & **c** were tested with two-sided t tests. p values presented in **d** & **e** were derived from ones-sided 50-repetition bootstrap tests (subspace and average relative angle different from 90°). Source data are provided as a Source Data file.

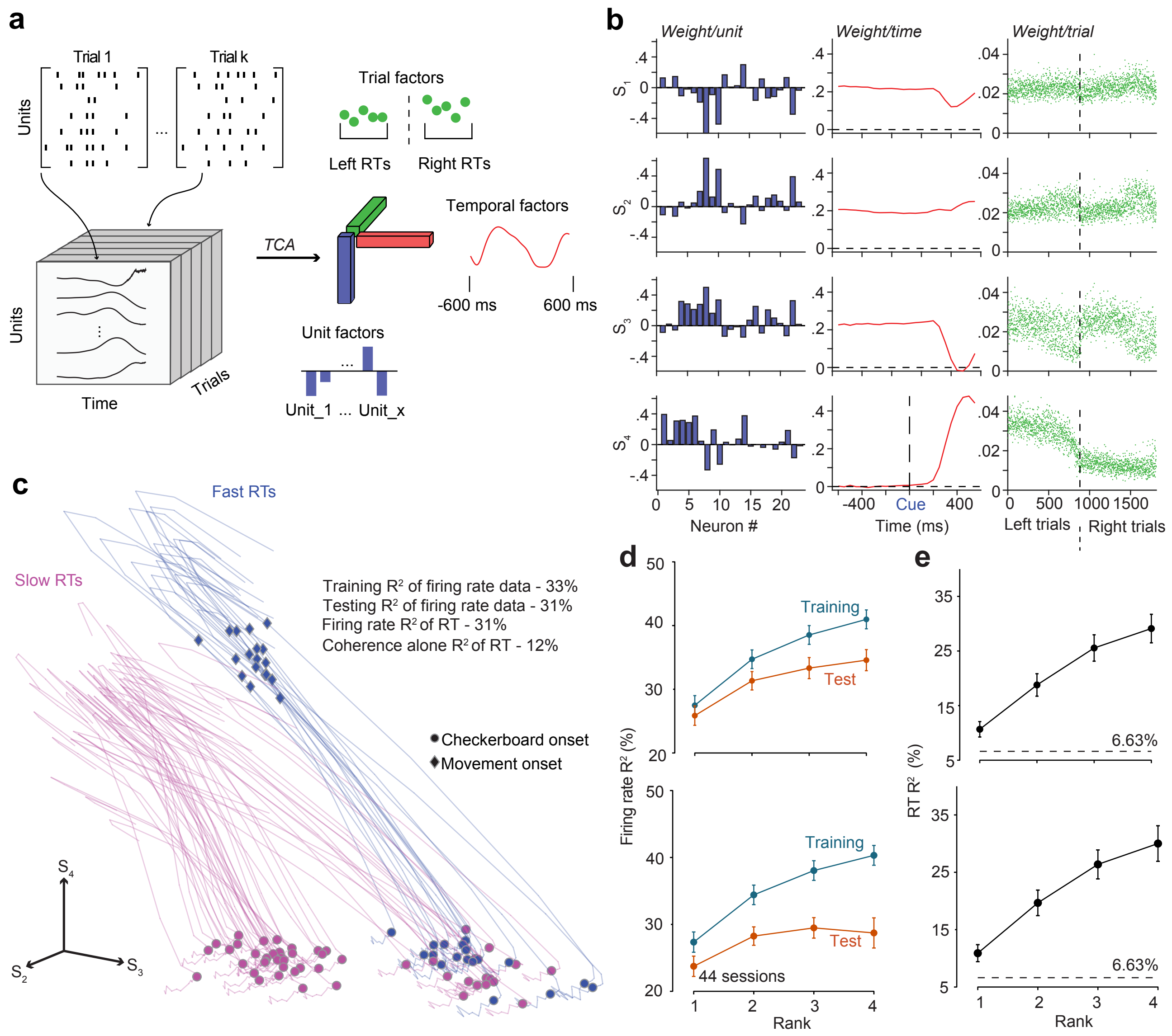

Figure S12: **Tensor component analysis recapitulates single-trial initial condition separation between slow and fast trials.** (**a**) Schematic for how tensor component analysis (TCA) is performed. TCA is a non-orthogonal linear dimensionality reduction technique that discovers the linear contribution of 3 tensors, in this case neuron, temporal, and trial factors (adapted from, Williams et al, 2018). (**b**) Weights for each of 23 units, time points (24) and trials (1814) across 4 factors (i.e., S_1, S_2, S_3, S_4) from a single session. Data are sorted by choice and RT for the trial factors. (**c**) Left-reach single-trial trajectories from a single session (same session as **b**, 23 units) in the space of the 2nd 3rd and 4th TCA factors (S_2, S_3, S_4). Projections are based on the product of S_2, S_3, and S_4 temporal weights with S_2, S_3, and S_4 trial weights. Thus these trajectories are an approximation of the neural population for slow (purple) and fast (blue) trials within this single session. Again, initial conditions separate as a factor of RT with the majority of slow RTs starting in a different state as compared to fast RTs. (**d**) Firing rate and (**e**) RT variance explained (R^2) for model fits with increasing rank (training set - blue and test set - orange). In **d** and **e** error bars are SEM from n = 44 sessions. Top: 25% of tensor data withheld (Speckled holdout) and Bottom: withhold a random neuron on 25% of trials. In **e**, Black dotted line is RT variance explained (6.63%) by linear regression model using coherence alone as the predictor. Source data are provided as a Source Data file.

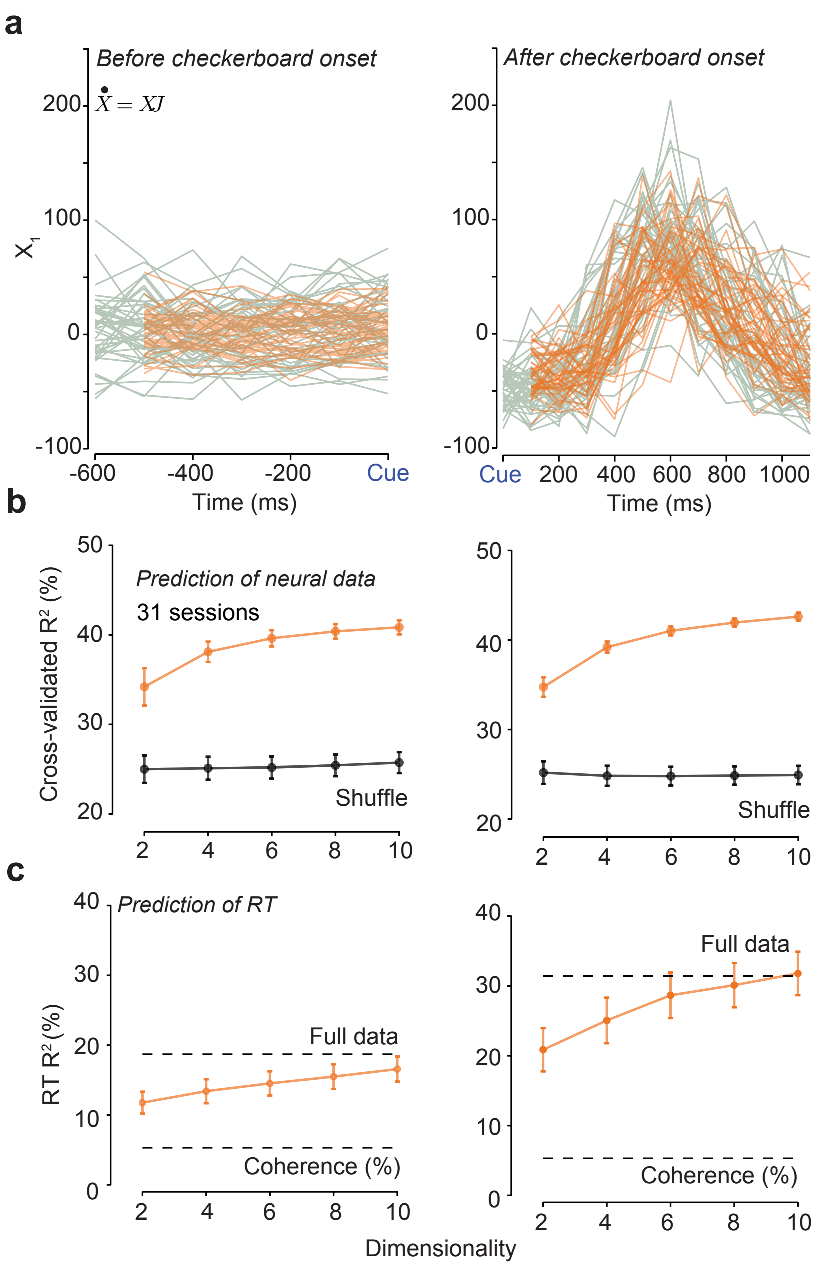

Figure S13: **Linear Dynamical Systems are a good model of neural activity, and are predictive of behavior.** (**a**) First latent variable of the LDS model before (left) and after (right) checkerboard onset. Gray lines depict held out neural data, whereas orange lines depict the held out neural data predicted at time point t by the equation $\overline{X}_{t-100ms}J$. J was learnt from a training set of trials and applied to a test-set. Note the close similarity between the predicted and real neural data. (**b**) LDSes of increasing dimensionality were built to model neural firing rates. The plots measure percent variance explained (R^2) of LDS-predicted firing rates as a function of increasing dimensionality for LDSes built from either neural data (orange) or its shuffle (gray). LDSes with greater dimensionality explain more of the neural variance as compared to a shuffle control before (left) and after (right) checkerboard onset. For the shuffle control, we randomized the time courses of neurons for each trial. (**c**) RT percent variance explained (R^2) from predicted neural activity from LDSes as a function of increasing dimensionality. Predicted neural activity from the LDS explains RT before (left) and after (right) checkerboard onset. Importantly, as dimensionality of the LDS increases, we can capture more neural activity (as shown in **b**), and also improve our ability to predict RT. In **b** and **c** error bars are SEM from n = 31 sessions. Source data are provided as a Source Data file.

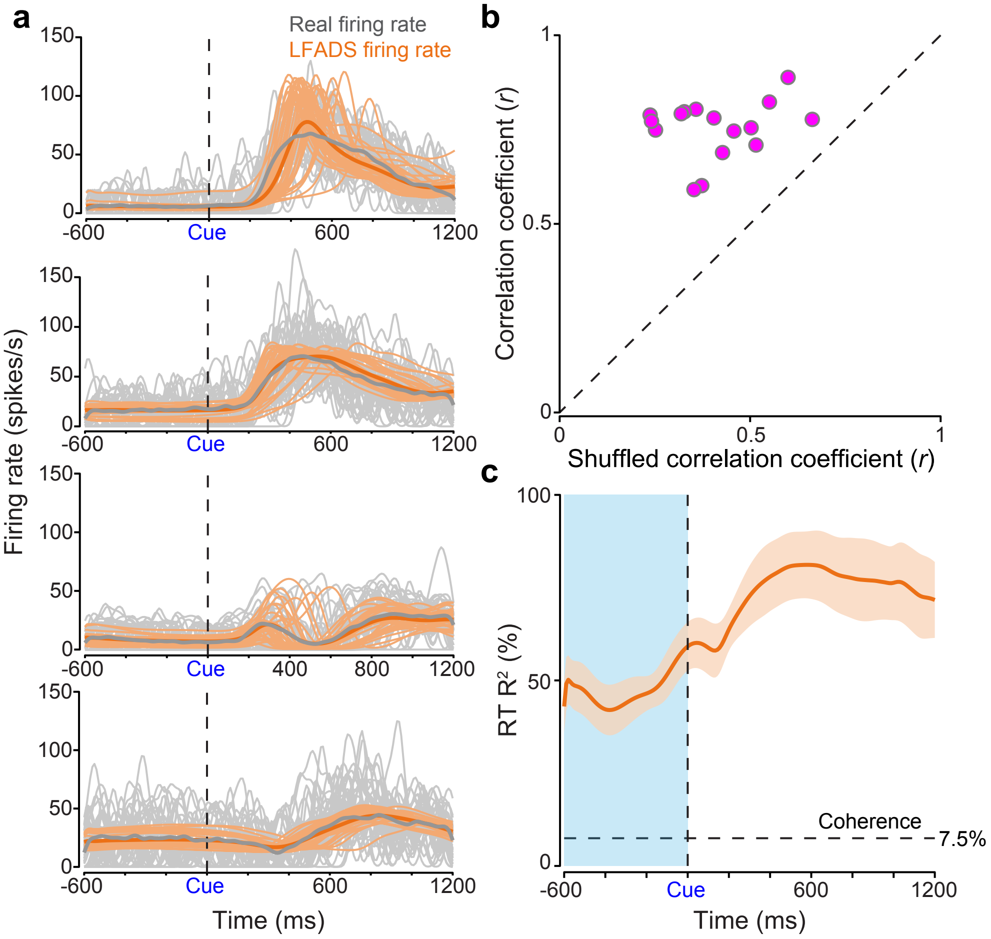

Figure S14: **LFADS analysis suggests that dynamical systems can recapitulate the firing rates of held out neural data and predict behavior.** (**a**) LFADS-predicted (orange) and real (gray; 20 ms Gaussian kernel) firing rates of 4 example units for all held-out left trials. (**b**) Scatter plot of the per session correlation coefficient (r) of LFADS-predicted firing rate correlated with real firing rate compared to the per session correlation coefficient of LFADS-predicted firing rate correlated with shuffled real firing rate. LFADS-predicted firing rates were correlated with 100 shuffles of real firing rates. The 99th percentile of correlation coefficients were used as the shuffled correlation coefficients. Dotted line indicates predicted r and shuffled r are the same. LFADS built on real neural data, predicts neural data better than the 99th percentile of the shuffled control in all sessions. (**c**) 16 session average of percent RT variance explained (R^2) from linear regressions using LFADS-predicted firing rates to predict RT. 7.5% is the variance explained by linear regression using stimulus coherence alone to predict RTs. Shaded error bars denote SEM. Source data are provided as a Source Data file.

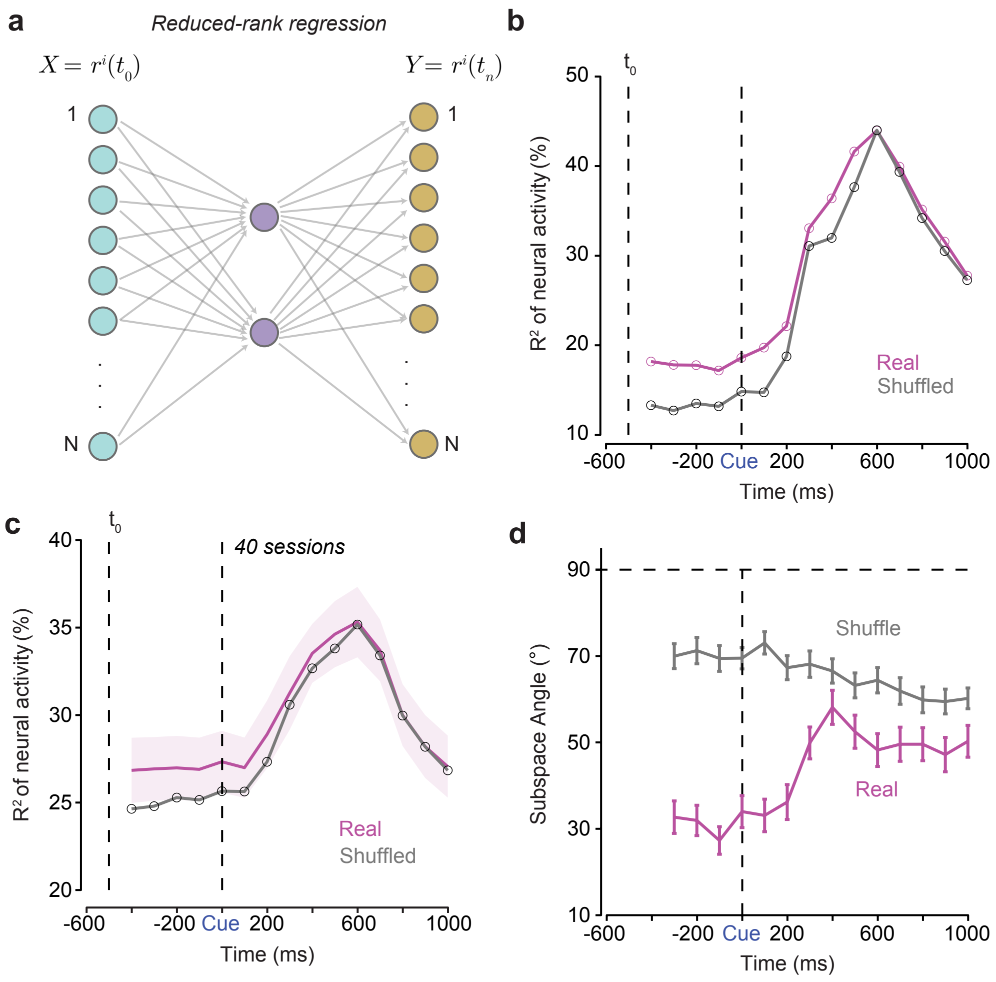

Figure S15: **Cross-validated reduced-rank regression shows that prestimulus state strongly predicts subsequent neural activity, and also RT.** (**a**) Schematic depicts reduced-rank regression where the neural data ($r^i(t_0)$, green dots) at time t_0 is fed into a reduced-rank predictor matrix (purple dots) to predict the neural data ($r^i(t_n)$, yellow dots) at time t_n. (**b**) Single-session cross-validated R^2 (trained on 70% of the data to estimate β and the rank of the predictor matrix) for real and shuffled neural data when using data from -550 ms before checkerboard onset as the neural data for the predictor matrix. We compared the predictions for the real data to a shuffled control where we randomized the neurons and again performed the regression. The predictor matrix included checkerboard coherence and choice, which explains the increasing amount of variance in neural activity that is accounted for even by the shuffled data after checkerboard onset. Before checkerboard onset, the intact data explains more of the neural activity than the shuffled data. (**c**) Average R^2 (41 sessions) of neural activity for real and shuffled data from reduced-rank regression. Again neural data explains more variance before checkerboard onset than shuffled neural data. Errorbars denote SEM. (**d**) Our KiNeT analysis with trial-averaged data suggested that the subspace was largely stable during the prestimulus period and started to change after stimulus onset when choice-related signals emerge (Fig. 4D, E). We repeated this analysis for the β estimated from reduced-rank regression at each time point. We calculated the angle between the β for the first time point to the β for all other time points (i.e., using the dot product formula to calculate angle between two vectors). We found that the angle between β was largely small and stable throughout the prestimulus period and into the poststimulus period. This again suggests a stable dynamical system from the prestimulus to poststimulus period until the emergence of choice signals (~ 200 ms). In contrast for the shuffle control, this angle was much higher suggesting that the β at each time point was not related to the β at other time points. Error bars are SEM from n = 41 sessions for **c**, **d**. Source data are provided as a Source Data file.

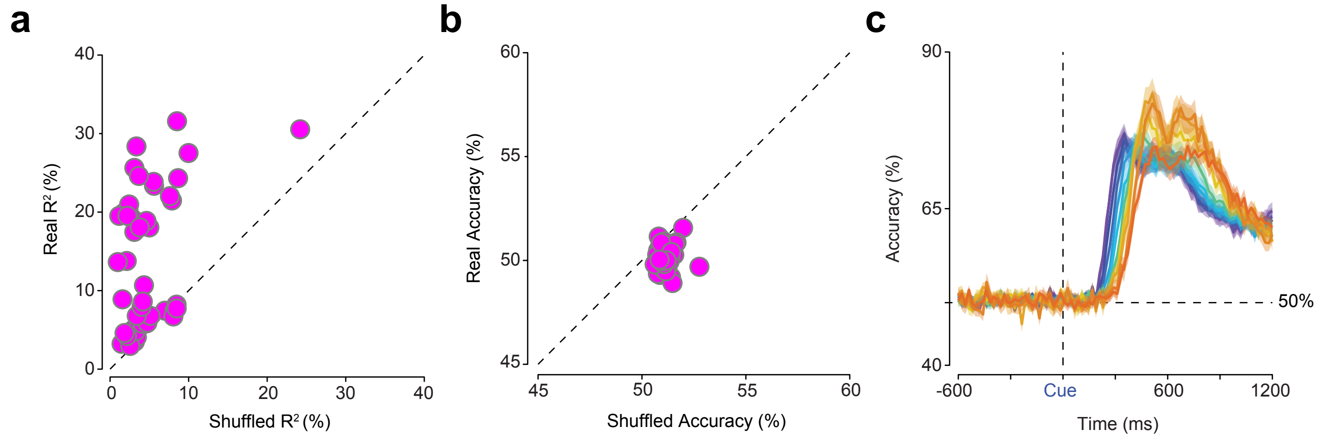

Figure S16: **Prestimulus spiking activity is predictive of RT but not choice, even when decoding is performed within RT bins** (**a**/**b**) Scatterplot of true mean prestimulus R^2/percent accuracy compared to the R^2/percent accuracy for the 99th percentile of the shuffled data. Each dot represents the bin- and trial-averaged prestimulus mean R^2/accuracy value within each of the 51 sessions. The dotted line is where scatter points would fall if shuffled R^2 and real R^2 values were equivalent. (**a**) Many of the points lie above this line suggesting that real prestimulus neural activity explains more of the RT variance than shuffled neural data. (**b**) In contrast, for choice many of the points lie on or below the 99% shuffle value suggesting that prestimulus neural activity is not predictive of choice. (**c**) Plot of mean accuracy from logistic regressions of binned spiking activity (20 ms) used to predict eventual choice within RT bins (number of trials was equalized for left and right choices). Accuracy is averaged across 51 sessions. Shaded area is SEM over these 51 sessions. The black dotted line is 50% accuracy. Data is pooled over all coherences. Source data are provided as a Source Data file.

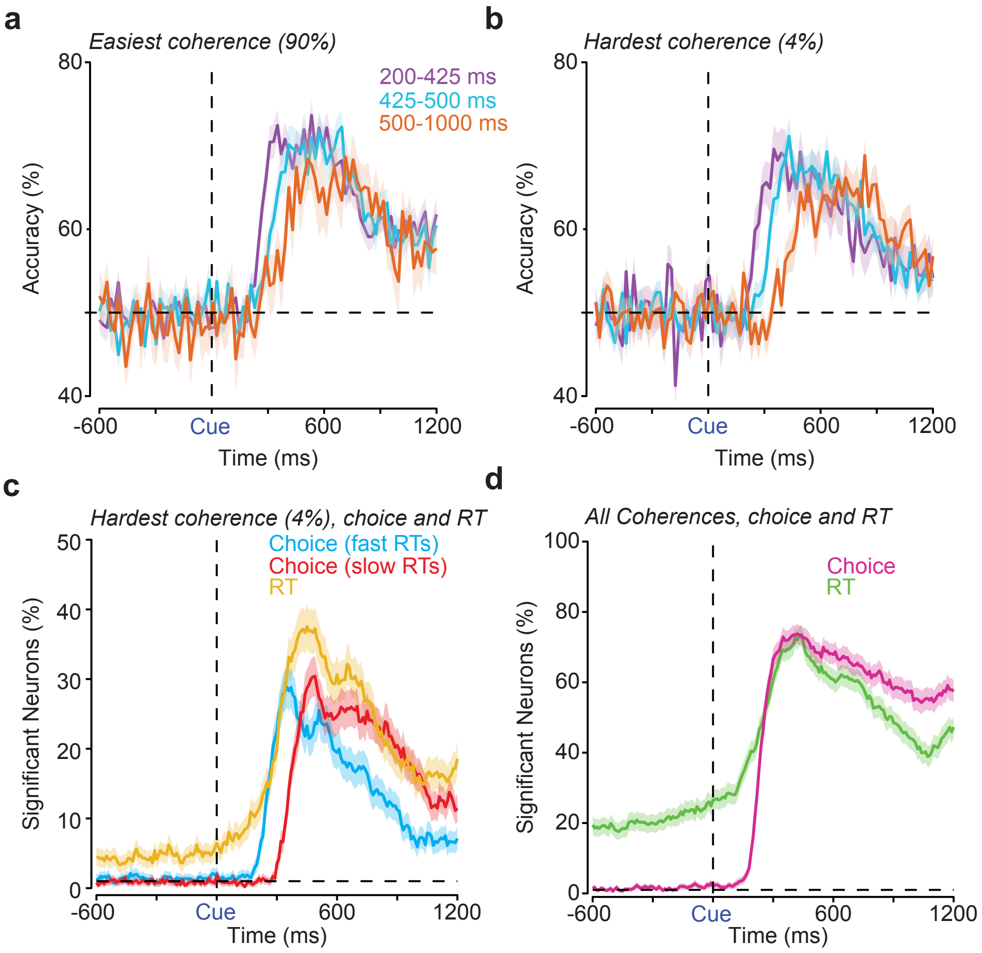

Figure S17: **Prestimulus spiking activity cannot predict choice, even when decoding is performed within RT bins for just the hardest coherence.** (**a, b**) Plot of mean accuracy from logistic regressions of binned spiking activity (20 ms) used to predict eventual choice within RT bins for the easiest (**a**) or the hardest coherence (**b**). Accuracy is averaged across 51 sessions. Color-matched shaded areas are SEM. The horizontal black-dotted line is 50% accuracy. RT bins for the decoding analysis are 200-425 ms (purple - fast), 425 - 500 ms (cyan - medium), 500 - 1000 ms (orange - slow). (**c**) Percent of units that covary with choice for only the hardest coherence and fast RTs (blue line) and slow RTs (red line), as measured using a regression model to predict choice. Each regression was performed separately. A neuron was assessed as having a significant relationship with choice if the 99% confidence interval did not overlap with 0. For both fast and slow RTs, the percent of units that show significant regression with choice before stimulus onset is largely at chance levels ($\sim$ 1%). However the percent of units that show significant regression with RT before stimulus onset is $\sim$5% from a separate regression with RTs, from only the hardest coherence, to predict neural activity (yellow line). (**d**) Percent of units that covary with choice (pink line) and RT (green line) when all coherences are included in the respective regressions. Again, prestimulus firing rates do not covary with choice but covary with RT. The coherence level of the stimulus was included with RT for this regression. Color-matched error bars are standard error of the percentage of units in **c** & **d** and are obtained through bootstrapping (1000 times over units). Source data are provided as a Source Data file.

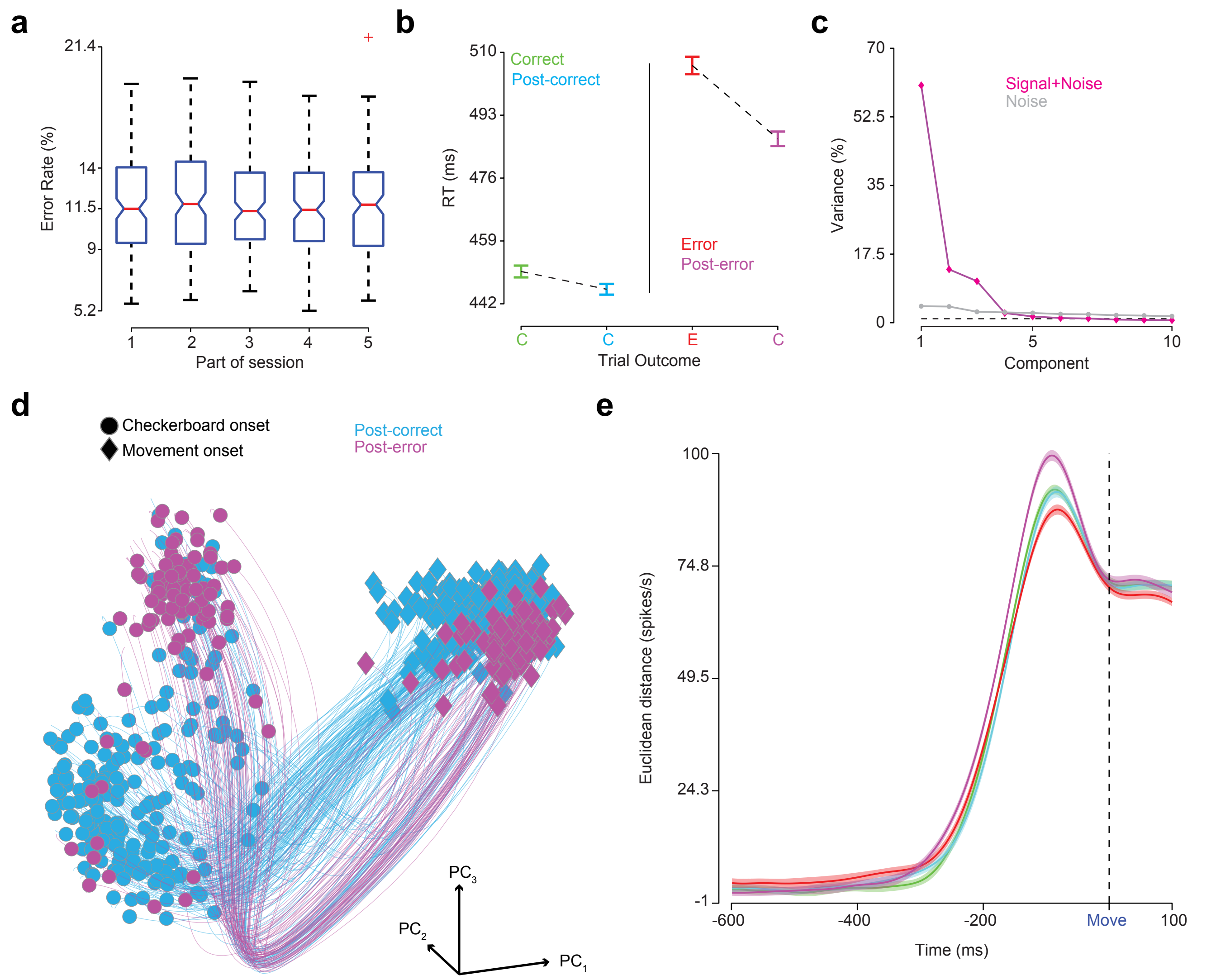

Figure S18: **Post-error correct trials are slower than post-correct correct trials.** (**a**) Boxplot of percentage of errors made in the session as a function of time in the session. Individual sessions (n = 141) varied in length and were thus divided into fifths based on number of trials per session (median number trials/session = 1,785). Center line is median, box limits are upper and lower quartiles, whiskers are 1.5x interquartile range, and outliers are plotted as red crosses. (**b**) Average RTs from all error, correct and trial-matched correct, correct sequences found across both monkeys and all sessions (error bars are $2 \times$ SEM). On average both monkeys demonstrate classical post-error slowing. RT data is pooled across both monkeys and only from trials (n = 17,512) where neural activity was simultaneously recorded. (**c**) Scree plot of the percentage of variance explained by the first ten components. The first six PCs capture $\sim 90\%$ of the variance in firing rate activity. (**d**) LFADS trajectories in the space of the first three orthogonalized factors ($PC_{1,2,3}$), obtained via PCA on LFADS latents, for 30% of post-correct and all post-error trials, for all coherences and left reaches from a single session (23 units). Each trajectory is plotted from 200 ms before checkerboard onset (dots) to movement onset (diamonds). (**e**) Average Euclidean distance in the first six dimensions, from 50 bootstrapped PCAs, between the two reach directions aligned to movement onset (Move & black dashed line). Color-matched shaded areas are 95% confidence intervals. We observe significantly more choice selectivity starting at least 250 ms before and, peaking ~ 90 ms before movement onset for post-error trials as compared to all other trial types. Choice selectivity is significantly lower ~ 90 ms before movement onset for error trials compared to all other trial types. Source data are provided as a Source Data file.

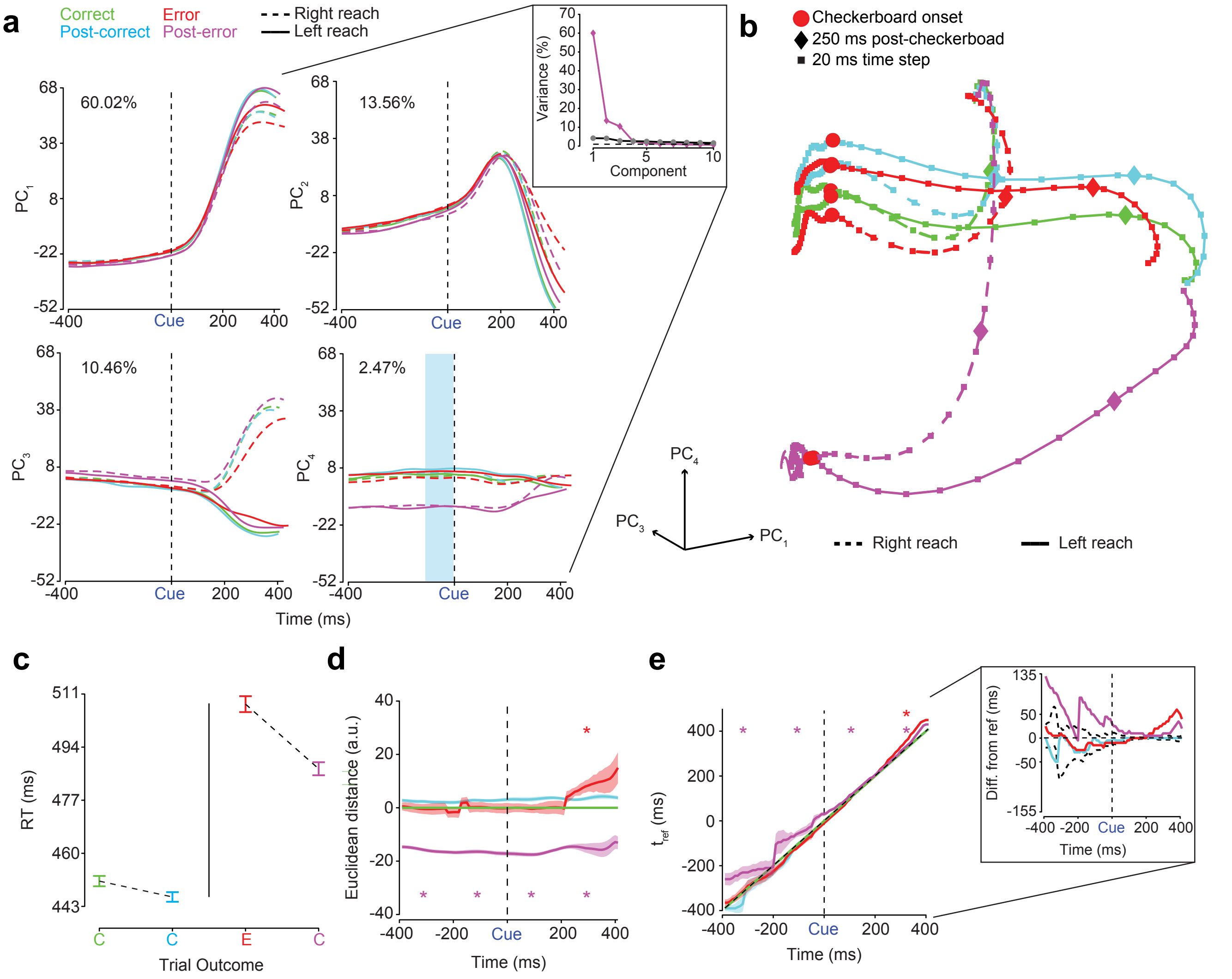

Figure S19: **Initial conditions covary with trial outcome even for alternative approaches.** To ensure that performance streaks do not bias the effects of trial outcome on prestimulus firing rates, we restricted our analysis to correct, correct, error (CCE) sequences and error, correct, correct (ECC) sequences. Results are nearly identical under such restrictions Fig. 8. (**a**) $PCs_{1,2,3,4}$ of trial-averaged firing rates aligned to checkerboard onset (Cue & black dashed line) organized by choice and trial outcome. Percentage variance is indicated at the top of each plot. *Inset*: Scree plot of the percentage of variance explained by the first ten components (pink, grey are noise components). The first six PCs capture $\sim 90\%$ of the variance in firing rate activity. (**b**) $PCs_{1,3,4}$ state space from 400 ms before to 400 ms after checkerboard onset (**c**) Average RTs from all EC and trial-matched CC sequences pooled across all sessions (error bars are $2 \times$ SEM) and indicating classical post-error slowing. RT data is only from trials (n = 15,486) where neural activity was simultaneously recorded. (**d**) KiNeT distance analysis demonstrating that trajectories are spatially organized with post-error trials furthest from other trial types peri-stimulus as compared to a reference trajectory (green, correct trials). (**e**) KiNeT Time to reference (t_{Ref}) analysis suggests slower prestimulus velocity for post-error trials as compared to the reference trajectory (green, correct trials). *Inset*: Difference from reference plotted against time. Data from post-correct, error, and post-error trials were subtracted from the reference trajectory (correct trials). Horizontal dotted line shows a difference of 0. Thicker dotted black lines show the 5th and 95th percentile of shuffled-data trajectories subtracted from the reference trajectory. In **d** & **e** the x-axis is labeled Time (ms), this should be understood as time in relation to the reference trajectory. Shaded regions, color matched to their respective trajectories, are bootstrap SEM. * - $p = 0.0196$. p values derived from one-sided 50-repetition bootstrap tests of differences between each outcome trajectory and the reference trajectory. *Abbreviation*: a.u. - arbitrary units. Source data are provided as a Source Data file.

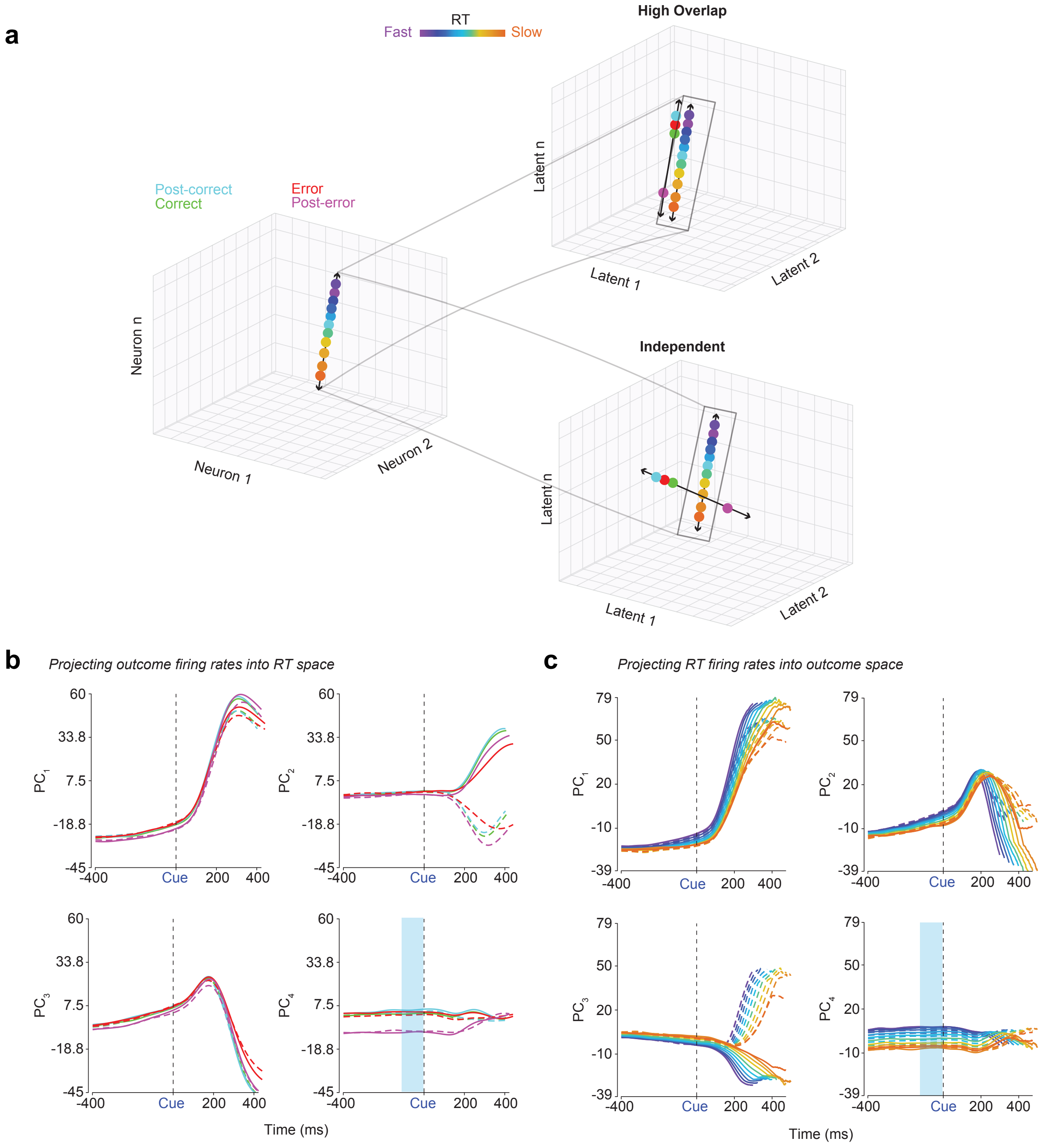

Figure S20: **Subspaces in PMd for RT and choice, and outcome and choice are overlapping.** (**a**) Schematic (adapted from, Elsayed et al, 2016) depicting the projection of firing rates organized by RT and choice onto the outcome subspace (first 6 principal components of firing rates organized by outcome and choice). One instance depicts high overlap (top) and the other depicts low overlap or independent case (bottom). (**b**) The first four PCs ($PC_{1,2,3,4}$) from the projection of trial-averaged firing rates organized by trial outcome and choice into a subspace (first 6 dimensions) identified by PCA on firing rates organized by RT and choice (Fig. 4) (**c**) The first four PCs ($PC_{1,2,3,4}$) from the projection of trial-averaged firing rates organized by RT and choice into a subspace (first 6 dimensions) identified by PCA on trial outcome and choice. Source data are provided as a Source Data file.

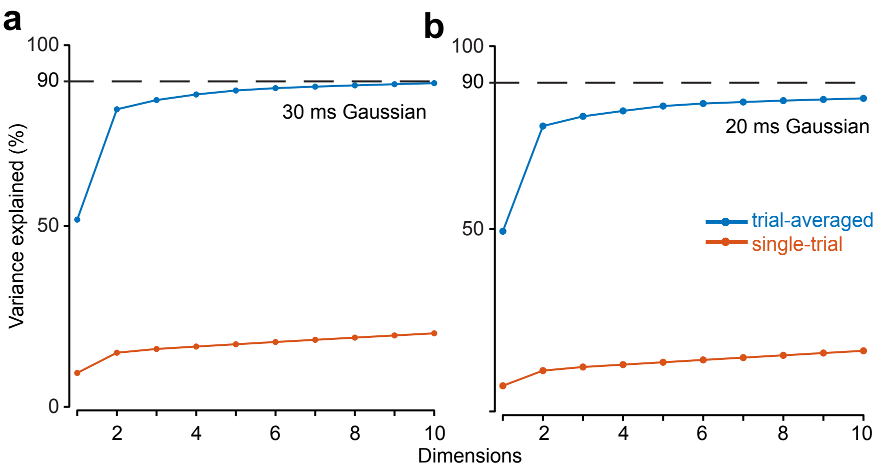

Figure S21: **Number of components to explain single trial variance is greater than the number of components needed for trial-averaged variance.** Variance explained by the first few components when PCA is performed on either the trial-averaged data from the simulated neural population in Fig. S3 by RT and choice, or on the single-trial firing rates. Spike trains are either smoothed by a 30 ms Gaussian (**a**) or by a 20 ms Gaussian (**b**). Note, trial-averaging suppresses spiking noise as well as single-trial variability leading to PCA needing far fewer dimensions to explain the data. In contrast, many more dimensions are needed to explain single-trial data because of variability induced by the latents as well as the spiking variability. Source data are provided as a Source Data file.

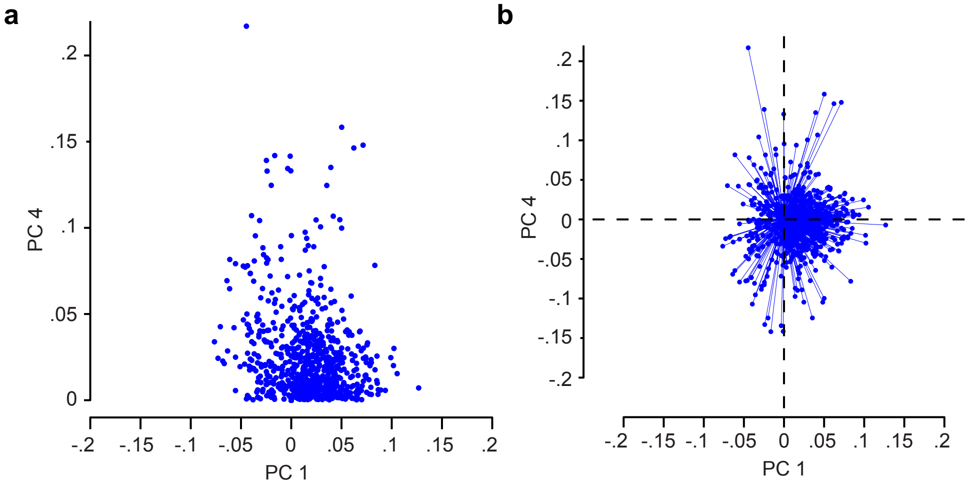

Figure S22: **PC loadings, from population of single units, do not reveal 'outcome'-specific subpopulation of units.** (**a**) Scatterplot of the loadings on the 1st PC and absolute value of the loadings for the 4th PC. (**b**) Biplot of loadings on the 1st PC and 4th PC. Source data are provided as a Source Data file.
