# Supplementary material for: Initial conditions combine with sensory evidence to induce decision-related dynamics in premotor cortex
Source: Nat Commun. 2023 Oct 16;14:6510. doi: 10.1038/s41467-023-41752-2 (PMC10579235; doi:10.1038/s41467-023-41752-2)
Supplement: Supplementary file 3 — Reporting Summary [file 41467_2023_41752_MOESM3_ESM.pdf]

Corresponding author(s): Dr. Chandramouli Chandrasekaran
Last updated by author(s): September 12th 2023

Reporting Summary

Nature Portfolio wishes to improve the reproducibility of the work that we publish. This form provides structure for consistency and transparency in reporting. For further information on Nature Portfolio policies, see our Editorial Policies and the Editorial Policy Checklist.

Statistics

For all statistical analyses, confirm that the following items are present in the figure legend, table legend, main text, or Methods section.

n/a	Confirmed	
☐	☒	The exact sample size (n) for each experimental group/condition, given as a discrete number and unit of measurement
☐	☒	A statement on whether measurements were taken from distinct samples or whether the same sample was measured repeatedly
☐	☒	The statistical test(s) used AND whether they are one- or two-sided<br>*Only common tests should be described solely by name; describe more complex techniques in the Methods section.*
☐	☒	A description of all covariates tested
☐	☒	A description of any assumptions or corrections, such as tests of normality and adjustment for multiple comparisons
☐	☒	A full description of the statistical parameters including central tendency (e.g. means) or other basic estimates (e.g. regression coefficient) AND variation (e.g. standard deviation) or associated estimates of uncertainty (e.g. confidence intervals)
☐	☒	For null hypothesis testing, the test statistic (e.g. F, t, r) with confidence intervals, effect sizes, degrees of freedom and P value noted<br>*Give P values as exact values whenever suitable.*
☒	☐	For Bayesian analysis, information on the choice of priors and Markov chain Monte Carlo settings
☒	☐	For hierarchical and complex designs, identification of the appropriate level for tests and full reporting of outcomes
☐	☒	Estimates of effect sizes (e.g. Cohen's d, Pearson's r), indicating how they were calculated

Our web collection on statistics for biologists contains articles on many of the points above.

Software and code

Policy information about availability of computer code

Data collection

Mathworks xPC target
Psychophysics toolbox
Cerebus system software (Blackrock)
Matclust (2015)
Plexon Offline Sorter

Data analysis

MATLAB (Various versions)
Statistics and Machine Learning Toolbox Version 12.2
    fitclinear function (using the Broyden-Fletcher-Goldfarb-Shanno quasi-Newton algorithm)
Curve Fitting Toolbox Version 3.6
Parallel Computing Toolbox

Tensor Decomposition (https://github.com/ahwillia/tensor-demo)
Kinematic Analysis of Variance (KiNeT; https://github.com/jazlab/KiNeT)
Tensor Toolbox for MATLAB v3.5 (Tensor Component Analysis; https://www.tensortoolbox.org/)
Latent Factor Analysis of Dynamical Systems (LFADS; https://github.com/lfads)
Reduced Rank Regression (https:// github.com/cmccomb/RedRank)
PsychRNN (Recurrent Neural Networks; https://github.com/murraylab/PsychRNN)
Linear dynamical systems (https://github.com/gamaleldin/CFR)
Demixed Principal Component Analysis (dPCA; https://github.com/machenslab/dPCA)

All code for replicating the figures and supplementary figures in the manuscript can be found at:

https://github.com/chand-lab/Dynamics2023/

For manuscripts utilizing custom algorithms or software that are central to the research but not yet described in published literature, software must be made available to editors and reviewers. We strongly encourage code deposition in a community repository (e.g. GitHub). See the Nature Portfolio guidelines for submitting code & software for further information.

Data

Policy information about availability of data

All manuscripts must include a data availability statement. This statement should provide the following information, where applicable:

- Accession codes, unique identifiers, or web links for publicly available datasets
- A description of any restrictions on data availability
- For clinical datasets or third party data, please ensure that the statement adheres to our policy

Source data are provided with this paper and can be used to recreate figures. Processed data used for generating figures have been deposited in Dryad [https://doi.org/10.5061/dryad.9cnp5hqn0]. Raw data are freely available upon request.

Research involving human participants, their data, or biological material

Policy information about studies with human participants or human data. See also policy information about sex, gender (identity/presentation), and sexual orientation and race, ethnicity and racism.

Reporting on sex and gender	N/A
Reporting on race, ethnicity, or other socially relevant groupings	N/A
Population characteristics	N/A
Recruitment	N/A
Ethics oversight	N/A

Note that full information on the approval of the study protocol must also be provided in the manuscript.

Field-specific reporting

Please select the one below that is the best fit for your research. If you are not sure, read the appropriate sections before making your selection.

☒ Life sciences ☐ Behavioural & social sciences ☐ Ecological, evolutionary & environmental sciences

For a reference copy of the document with all sections, see nature.com/documents/nr-reporting-summary-flat.pdf

Life sciences study design

All studies must disclose on these points even when the disclosure is negative.

Sample size	We used two macaques in this study. Study design was based on the best practices in the world and in basic neuroscience studies involving macaques (see our, and other papers by Mante, Sussillo, Shenoy, Newsome, 2013, or Roitman and Shadlen 2002). However this study is overpowered in number of sessions that each macaque performed (T -75 sessions, O - 66 sessions), trials (each subject performed over 100,000 behavioral trials where neural inferences were used) and units recorded from (996 units). All conclusions about neural population dynamics are drawn from the 996 units that were recorded from across both animals and multiple sessions.
Data exclusions	No data were excluded in this study.
Replication	No explicit replication was performed for our study. Replicating the near 151 sessions used in our dataset in 2 new monkeys would entail nearly 5 years of work. However, we should note that our central findings are supported or replicated using different analysis techniques, suggesting robust findings. Moreover, although not conducted in the same lab, some aspects of our findings were reported in related studies suggesting again the robustness of our findings.
Randomization	Randomization into separate groups is not applicable in this study as there were no groups; both animals performed the same task under the same conditions, over many sessions and trials. However, all trials were randomized as were placement of electrodes on a day to day basis. Checkerboard stimuli were drawn from a random distribution.
Blinding	Blinding is not relevant to this study as the macaques were not placed into different groups and the same task under the same conditions was performed from session-to-session.

Reporting for specific materials, systems and methods

We require information from authors about some types of materials, experimental systems and methods used in many studies. Here, indicate whether each material, system or method listed is relevant to your study. If you are not sure if a list item applies to your research, read the appropriate section before selecting a response.

Materials & experimental systems

n/a	Involved in the study
☒	☐ Antibodies
☒	☐ Eukaryotic cell lines
☒	☐ Palaeontology and archaeology
☐	☒ Animals and other organisms
☒	☐ Clinical data
☒	☐ Dual use research of concern
☒	☐ Plants

Methods

n/a	Involved in the study
☒	☐ ChIP-seq
☒	☐ Flow cytometry
☒	☐ MRI-based neuroimaging

Animals and other research organisms

Policy information about studies involving animals; ARRIVE guidelines recommended for reporting animal research, and Sex and Gender in Research

Laboratory animals	2 male macaque mulatta. T - 7 years old, O -11 years old.
Wild animals	The study did not involve wild animals.
Reporting on sex	Both macaques were males. Female macaque monkeys are difficult to obtain. The small sample size in such studies also precludes any statements about sex differences.
Field-collected samples	The study did not involve samples collected from the field.
Ethics oversight	Stanford University Institutional Animal Care and Use Committee approved the study protocol (8856).

Note that full information on the approval of the study protocol must also be provided in the manuscript.
